# Leishmaniosis in Rodents Caused by *Leishmania infantum*: A Review of Studies in the Mediterranean Area

**DOI:** 10.3389/fvets.2021.702687

**Published:** 2021-08-06

**Authors:** M. Magdalena Alcover, M. Cristina Riera, Roser Fisa

**Affiliations:** Section of Parasitology, Department of Biology, Health, and Environment, Faculty of Pharmacy and Food Science, University of Barcelona, Barcelona, Spain

**Keywords:** *Leishmania infantum*, zoonosis, rodentia, reservoir, wildlife, Mediterranean basin

## Abstract

Leishmaniosis infection begins when a phlebotomine sand fly vector inoculates pathogenic protozoan parasites of the genus *Leishmania* into a mammalian host. In the case of *Leishmania infantum*, the domestic dog is considered to be the main parasite reservoir, and canine leishmaniosis (CanL) has a high mortality rate in untreated dogs. Hundreds of cases of human leishmaniosis (HL) are reported in the world each year, the incidence in Europe being relatively low. Leishmaniosis control is primarily focused on the dog, combining methods that prevent sand fly bites and boost host resistance to infection. However, these measures are only partially effective and new solutions need to be found. One of the main factors limiting CanL and HL control is the existence of a sylvatic *Leishmania* transmission cycle that interacts with the domestic cycle maintained by dogs. It is suspected that the main reservoir of infection in wildlife are rodents, whose expansion and rapid population growth worldwide is increasing the risk of human and zoonotic pathogen transfer. The aim of this review is therefore to analyze reports in the literature that may shed light on the potential role of rodents in the leishmaniosis transmission cycle in the Mediterranean area. Following the general methodology recommended for reviews, six databases (Google Scholar, Ovid, PubMed, Science Direct, Scopus and Web of Science) were explored for the period January 1995 to December 2020. The results extracted from 39 publications that met the established inclusion criteria were analyzed. It was found that 23 species of rodents have been studied in nine countries of the Mediterranean basin. Of the 3,643 specimens studied, 302 tested positive for *L. infantum* infection by serology, microscopy and/or molecular techniques.

## Introduction

Leishmaniosis is a parasitic vector-borne disease caused by *Leishmania* spp. affecting humans and other mammals. In Europe, leishmaniosis (caused mainly by *Leishmania infantum*) is an emerging zoonosis, with 700 new cases appearing annually (1). Of the four *Leishmania* species present in the Mediterranean basin, *L. infantum* is predominant and the causative agent of the human form of visceral (VL), cutaneous (CL), and mucocutaneous leishmaniosis (MCL). The others are *Leishmania major* (North Africa and the Middle East; CL), *Leishmania tropica* (Greece, Turkey, the Middle East and North Africa; CL), and *Leishmania donovani* (Cyprus; VL and CL) (2, 3).

These *Leishmania* species are capable of spreading to new geographical areas that have sufficient numbers of suitable sand fly vectors and favorable ecological conditions (4).

In the Mediterranean basin and surrounding areas, where *L. infantum* is endemic (5–8), dogs are currently considered to be the main reservoir. Thanks to the application of molecular tools and serology, *Leishmania* has been detected in clinically healthy and seronegative mammals, not only dogs but also other domestic/peridomestic and wild mammal species, including rodents (9–13). The long list of potential reservoir hosts suggests that *Leishmania* can be transmitted to a diverse range of mammals through sand fly bites and that wild mammals can suffer frequent and non-specific infection (14). The abundance and widespread distribution of rodents, together with their longevity, which allows them to survive an entire sand fly season, makes them likely candidates for infection with *Leishmania* species, including *L. infantum* (15). Furthermore, rodents are known to remain asymptomatic carriers for very long periods (16–19). Consequently, it can be hypothesized that rodent populations, as well as other wild animals, can maintain the permanent circulation of the parasite in an endemic area.

Many interacting host species fulfill the criteria that define a reservoir of *Leishmania* (abundance; attracting and infecting sand flies; evidence of long-term infection at the individual or species level) (20). Nevertheless, their categorization as primary, secondary, or accidental reservoirs depends on local ecological and epidemiological conditions. Although, it has not been demonstrated that rodents (and wildlife in general) act as a reservoir for *Leishmania*, some species of rodents are known to contribute to maintaining the circulation of *L. infantum* in certain areas of southern Europe (11). Studies employing xenodiagnosis could help to determine the role of wildlife in the current epidemiology of leishmaniosis, as demonstrated in an outbreak in Fuenlabrada (Madrid) (21), where this approach incriminated lagomorphs as the source of human infection spread by phlebotomine sand flies. Unfortunately, these types of studies are difficult to carry out in wildlife.

The aim of this review is to provide an overview of studies dealing with the potential role of rodents in the life cycle of *L. infantum* and the current epidemiological status of leishmaniosis in the Mediterranean basin.

## Materials and Methods

### Search and Eligibility Criteria

A bibliographic search was carried out in the databases of Google Scholar, Ovid, Pubmed, ScienceDirect, Scopus, and Web of Science. The general terms “leishmania infantum,” “epidemiology,” and “detection” were used, together with the MeSH term “rodentia.” If the latter was not accepted, it was replaced by the general term “rodent.” The selected articles were those dealing with studies on rodent species as leishmaniosis reservoirs in the Mediterranean area. Other inclusion criteria were the language (English) and date of publication (between January 1, 1995 and December 31, 2020). This review was carried out essentially based on guidelines outlined in the study published in Research Synthesis Methods (22).

Restricting the review to studies published in English may be considered a limitation, as the Mediterranean area has a wide diversity of languages. Nevertheless, as all the studies included here have been published in indexed journals, their rigor is ensured. We have also referenced the four articles found in the search that have an abstract in English but were excluded from the review as otherwise they are written in Turkish (one) (23) and French (one) (24).

### Statistics

The SPSS program, version 25.0 (SPSS, Chicago, IL, USA), and the GraphPad Prism Software program, version 8.0 (La Jolla, CA, USA) were used for the different statistical analyses performed. The normality of the different variables studied was verified with the Kolmogorov-Smirnov test (*P* ≥ 0.200). The differences in medians were compared using the Mann-Whitney U test (for two independent variables) or the Kruskal-Wallis test (for more than two independent variables), because they were non-parametric variables. The frequencies of the different variables studied were compared using the *X*^2^ test. In all cases, *P*-values < 0.05 were reported as statistically significant.

## Results and Discussion

A total of 39 articles were included for review (11, 13, 14, 21, 25–59). The number of rodents examined in all the reported studies was 3,643, 302 of which were infected, implying an infection prevalence of 8.3%, (IC_95%_ 7.4–9.2) (Supplementary Data Sheets 1, 2). Infection was detected in different sample types using molecular techniques, and only four studies also included serological methods [Dabaghmanesh et al. (29), Tsakmakidis et al. (59), Othman et al. (27), Alcover et al. (13)].

**Figure 1:**
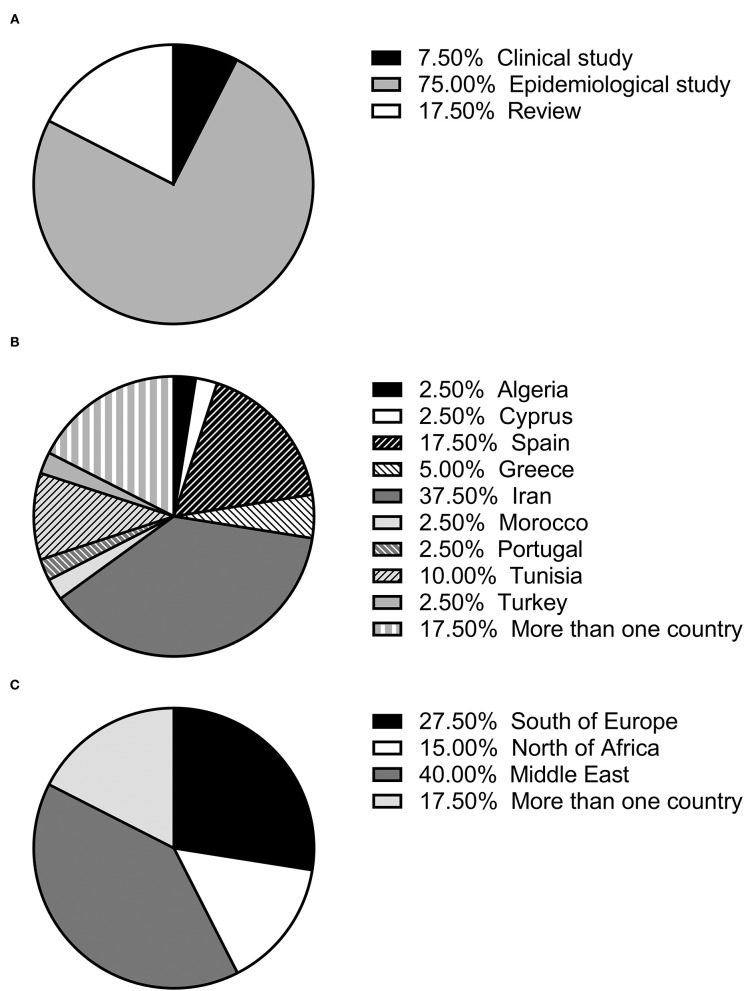
**(A)** Percentage of each type of study (review, clinical or epidemiological); **(B)** percentage of studies published in each country; **(C)** percentage of studies published in each geographical area.

The selected articles were classified as clinical, epidemiological and review studies, the great majority (75%) being epidemiological (Figure 1A). In the last 25 years, the possible role of rodents in the *L. infantum* cycle has been assessed in nine countries of the Mediterranean basin, with the highest number of studies being carried out in Iran. Seven studies were performed in more than one of these nine countries (11, 14, 25, 41, 44, 45, 52) (Figure 1B). If the Mediterranean basin is divided into three large geographical areas, Southern Europe, North Africa and the Middle East, the latter concentrates the greatest number of studies (Figure 1C).

**Figure 2:**
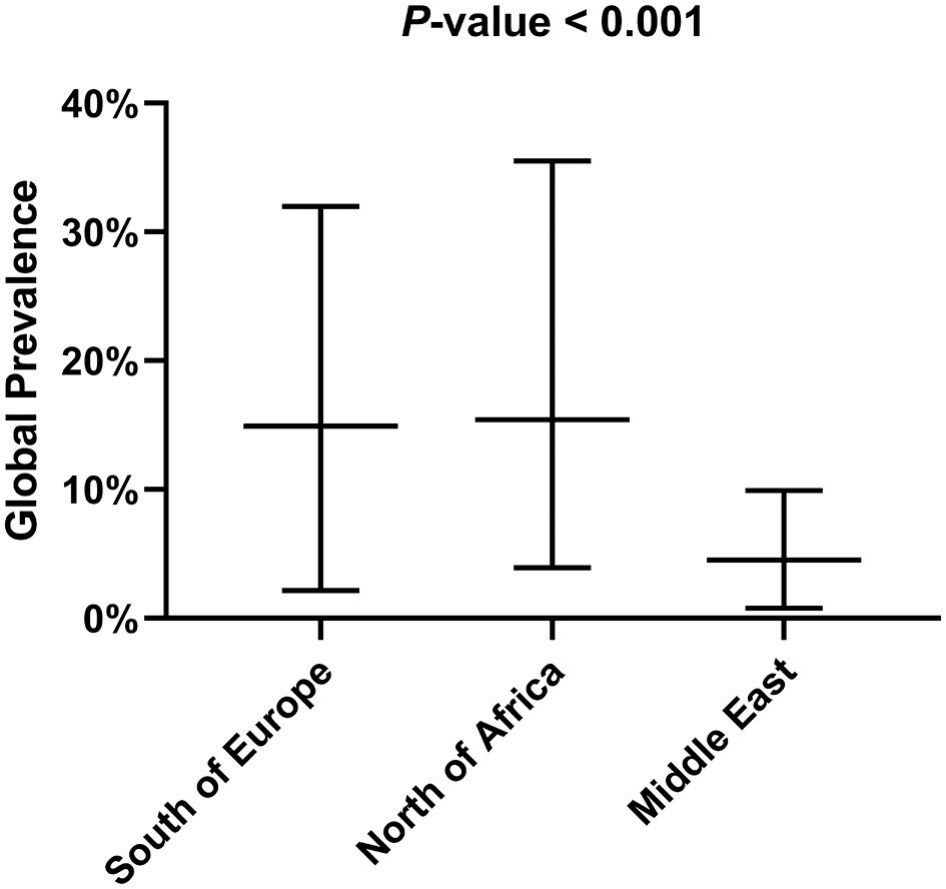
Global prevalence of infection in rodents depending on the geographical area.

Tables 1, 2 show the number of animals studied in each country and geographical area, as well as the number of infected specimens and prevalence of infection. Statistically significant differences in the proportion of infected hosts were found only between geographical areas, with the highest rate in Southern Europe, above all in Portugal and Spain (more than 25%). When considering the overall prevalence of infection in rodents, a significant difference (*P*-value of Chi-Square < 0.001) is again apparent between the Middle East (4.5%), North Africa (15.4%), and Southern Europe (14.9%) (Figure 2). The differences observed between these geographical areas may be due to a variable diversity of *Leishmania* species (25, 32), and that rodents have been reported to host more than one species (26). When drawing conclusions from the data reviewed here, it should therefore be taken into account that an animal may be parasitized by more than one species of *Leishmania* or that identification at species level could be absent.

**Table 1:** Median (rank) of studied rodents and the prevalence of infection in each country.

	**Number of rodents**	***P*-value**	**Number of infected rodents**	***P*-value**	**Prevalence of infection**	***P*-value**
Country
Algeria	3 (-)	0.150	0 (-)	0.369	0.00 (-)	0.131
Cyprus	494 (-)		36 (-)		7.29 (-)	
Spain	37 (7–150)		9 (1–29)		29.59 (0.67–88.89)	
Greece	57 (16–97)		14 (1–19)		16.53 (6.25–26.80)	
Iran	108 (15–566)		1 (0–60)		0.27 (0.00–30.93)	
Morocco	197 (-)		16 (-)		8.12 (-)	
Portugal	30 (-)		9 (-)		30.00 (-)	
Tunisia	72 (-)		26 (-)		36.11 (-)	
Turkey	432 (-)		5 (-)		1.16 (-)	

*P-value ≤ 0.05 is considered as statistically significant*.

**Table 2:** Median (rank) of studied variables according to the geographical area.

	**Number of rodents**	***P*-value**	**Number of infected rodents**	***P*-value**	**Prevalence of infection**	***P*-value**
Area
Southern Europe	37 (7–494)	0.168	9 (1–36)	0.150	26.80 (0.67–88.89)	0.020
North Africa	72 (3–197)		16 (0–26)		8.12 (0.00–36.11)	
Middle East	117 (15–566)		3 (0–60)		0.72 (0.00–30.93)	

*P-value ≤ 0.05 is considered as statistically significant*.

**Figure 3:**
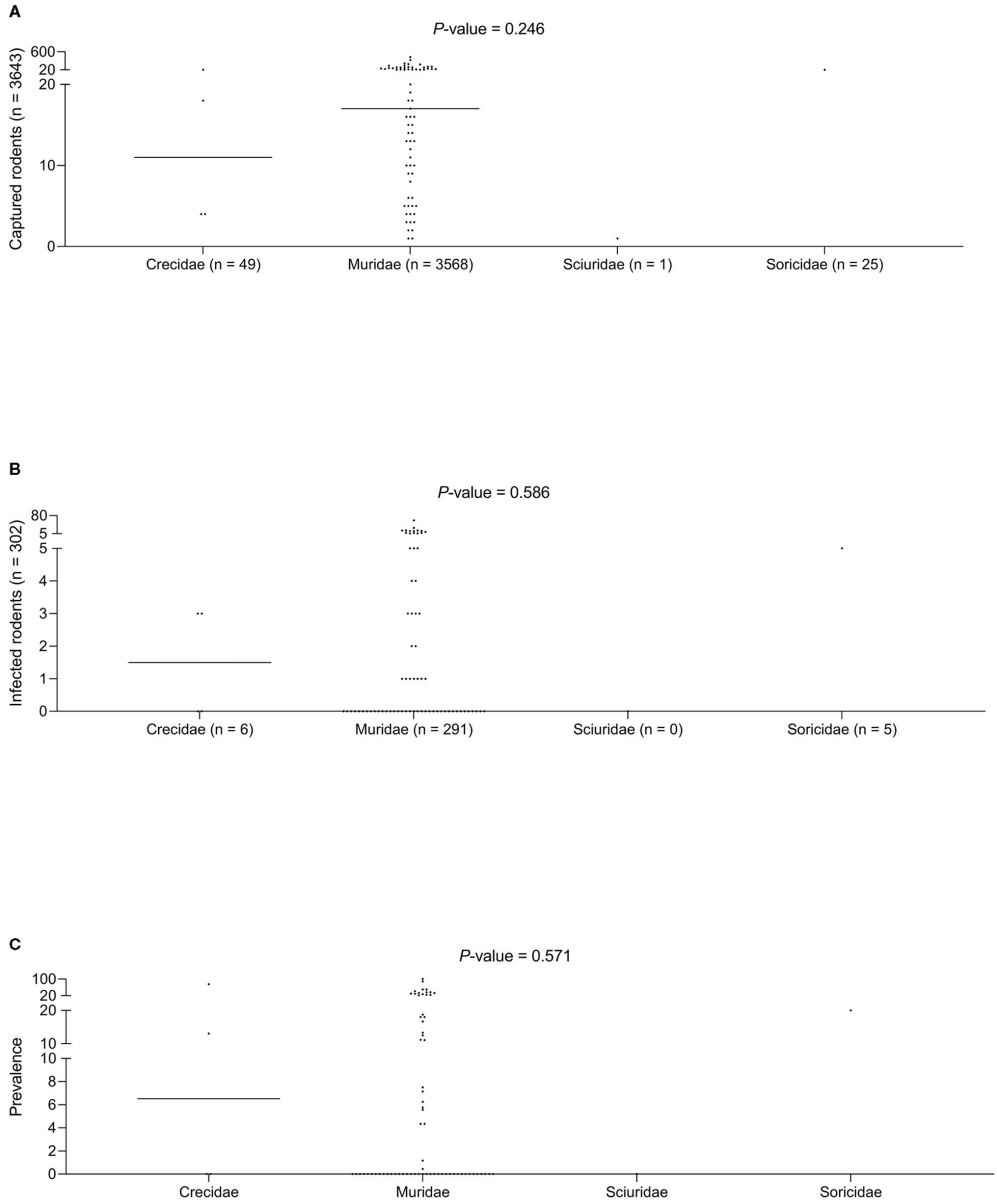
**(A)** Captured rodents. **(B)** Infected rodents. **(C)** Prevalence of infection among different rodent families.

No statistically significant differences were found for the three variables studied (the number of animals captured, number of infected animals, and the median prevalence of infection for each family/subfamily/species) (Figures 1, 3). However, when analyzing the overall prevalence, statistically significant differences become apparent for each species and subfamily, as shown in Table 3. The highest overall infection rate is found in *Mesocricetus auratus* (44), whereas, 10 of the 25 studied species have 0% infection.

**Table 3:** Global prevalence and IC_95%_ of infection in each species and subfamily.

**Subfamily**	**Species**	**Global prevalence of infection**	**IC_**95%**_**	***P*-value**	***P*-value**
Arvicolinae		0.0%	–		<0.001
	*Microtus arvalis*	0.0%	–	–	
Callosciurinae		0.0%	–		
	*Funambulus pennantii*	0.0%	–	–	
Cricitinae		15.0%	3.93–26.07%		
	*Cricetulus migratorius*	8.3%	2.4–20.6%	<0.001	
	*Mesocricetus auratus*	75%	28.4–97.2%		
Gerbillinae		2.98%	2.08–3.88%		
	*Gerbillus campestri*	0.0%	–	<0.001	
	*Meriones crassus*	0.0%	–		
	*Meriones hurrianae*	0.0%	–		
	*Meriones libycus*	1.1%	0.4–2.3%		
	*Meriones persicus*	6.5%	3.0–12.4%		
	*Meriones shawi*	0.0%	–		
	*Psammomys obesus*	39.1%	26.0–53.5%		
	*Psammomys vexillaris*	27.6%	14.0–45.4%		
	*Rhombomys opimus*	0.3%	0.0–1.2%		
	*Tatera indica*	0.8%	0.2–2.7%		
Murinae		11.45%	10.11–12.78%		
	*Apodemus sylvaticus*	2.2%	1.2–3.5%	<0.001	
	*Lemniscomys barbarus*	0.0%	–		
	*Mastomys erythroleucus*	0.0%	–		
	*Mus musculus*	16.6%	12.8–20.9%		
	*Mus spretus*	19.4%	11.6–29.7%		
	*Nesokia indica*	0.0%	–		
	*Rattus norvegicus*	16.4%	14.0–19.0%		
	*Rattus rattus*	9.9%	6.9–13.6%		
Sciurinae		20.0%	8.4–39.6%		
	*Sciurus vulgaris*	20.0%	8.4–39.6%	–	

*A P-value ≤ 0.05 is considered statistically significant*.

In studies carried out in Southern Europe published between January 2001 and December 2020 (Tables 4, 5), six rodent species were examined in five countries. No statistically significant differences were found related to the different types of sample analyzed (blood, bone marrow, hair, liver, skin, and spleen).

**Table 4:** Median and rank of infection prevalence in the different tissue samples analyzed, the number of positive samples and th number of analyses in Southern Europe between January 2001 and December 2020.

**Parameter**	**Median**	**Rank**	***P*-value**
**Prevalence of infection (%)**			
Blood	18.09	5.52 – 70.00	0.854
Bone marrow	18.06	8.33 – 44.44	
Leg hair	33.33	–	
Liver	19.68	12.00 – 33.33	
Skin	16.67	8.33 – 66.67	
Spleen	13.75	4.17 – 33.33	
More than one sample	18.75	0.00 – 100.00	
**Number of positive samples**			
Blood	10	1 – 19	0.545
Bone marrow	2	1 – 8	
Leg hair	2	–	
Liver	3	2 – 6	
Skin	2	1 – 12	
Spleen	3	1 – 29	
More than one sample	1	0 – 16	
**Number of analyses**			
Blood	18	4 – 344	0.587
Bone marrow	14	4 – 24	
Leg hair	6	–	
Liver	17	9 – 35	
Skin	18	4 – 35	
Spleen	24	9 – 102	
More than one sample	18	2 – 66	

*P-value ≤ 0.05 is considered as statistically significant*.

**Table 5:** Studies carried out in Europe published between January 1 2001 and December 31 2020.

**Species of rodentia**	**Ref**.	**Country**	**Sample (positive animals/total animals)**	**Detection technique**	**% of positivity (IC_**95%**_)**
*Apodemus sylvaticus*	Navea-Pérez et al. (47)	Spain (Granada)	Bone marrow (2/24) 8.33% (0.00–19.39) Spleen (1/24) 4.17% (0.00–12.16) Skin (2/24) 8.33% (0.00–19.39)	PCR-ELISA	20.8 (4.56–37.04) *N* total detected = 5
	Risueño et al. (21)	Spain (Murcia)	Liver/Spleen/Skin (3/16) 18.75% (0.00–37.88)	Real-time PCR	18.8 (0.00–37.94) *N* total detected = 3
	Ortuño et al. (48)	Spain (Murcia)	Liver/Spleen/Skin (1/2) 50.00 (0.00–100.0)	Real-time PCR	50 (0.00–100.00) *N* total detected = 1
*Mus musculus*	Helhazar et al. (36)	Portugal (Sesimbra)	Skin (9/27) 33.33%% (15.55–51.11)	qPCR	33.3 (15.52–51.08) *N* total detected = 9
			Liver/Spleen (8/27) 29.63% (12.41–46.85)	Microscopy (Giemsa/HE)	
	Navea-Pérez et al. (47)	Spain (Granada)	Bone marrow (1/4) 25.00% (0.00–67.44) Blood (1/4) 25.00 (0.00–67.44) Skin (1/4) 25.00 (0.00–67.44)	PCR-ELISA	50 (1.00–99.00) *N* total detected = 2
	Tsakmakidis et al. (59)	Greece (Macedonia)	Blood/Liver/Spleen (16/66) 24.24% (13.90–34.58)	qPCR	24.2 (13.87- 34.53) *N* total detected = 16
			Liver/Spleen (0/66) 0.00 (0.00–0.00)	Microscopy	
			Blood (13/26) 50.00 (30.78–69.22)	ELISA	
	Martín-Sánchez et al. (39)	Spain (Granada)	Bone marrow (8/18) 44.44% (21.49–67.40) Spleen (1/18) 5.56% (0.00–16.14) Skin (12/18) 66.67% (44.89–88.44)	PCR-ELISA, qPCR	88.9 (74.39–100.00) *N* total detected = 16
*Mus spretus*	Millán (48)	Spain (Barcelona)	Blood/Spleen(1/23) 4.35% (0.00–12.68)	Real-time PCR	4.3 (0.00–12.59) *N* total detected = 1
	Alcover et al. (13)	Spain (Catalonia)	Liver (6/35) 17.14% (4.66–29.63) Skin (5/35) 14.29% (2.69–25.88) Spleen (10/35) 28.57% (13.60–43.54)	qPCR	42.9 (26.50–59.30) *N* total detected = 9
*Rattus norvegicus*	Psaroulaki et al. (53)	Cyprus	Blood (19/344) 5.52% (3.11–7.94)	IFAT	5.5 (3.09–7.91) *N* total detected = 19
	Papadogiannakis et al. (50)	Greece (Athens and Piraeus)	Spleen (1/16) 6.25% (0.00–18.11)	nPCR	6.3 (0.00–18.21) *N* total detected = 1
	Tsakmakidis et al. (59)	Greece (Macedonia)	Blood (7/10) 70.00% (41.60–98.40)	ELISA	70 (41.60–98.40) *N* total detected = 7
			Liver/Spleen (0/18) 0.00% (0.00–0.00)	Microscopy	
			Liver/Spleen (0/18) 0.00% (0.00–0.00)	PCR	
	Helhazar et al. (36)	Portugal (Sintra)	Liver (3/9) 33.33% (2.53–64.13) Spleen (3/9) 33.33% (2.53–64.13)	Microscopy	33.3 (2.51–64.09) *N* total detected = 3
	Muñoz-Madrid et al. (60)	Spain (Extremadura)	Hair of legs (2/6) 33.33% (0.00–71.05)	Real-time PCR	33.3 (0.00–71.01) *N* total detected = 2
	Ortuño et al. (48)	Spain (Murcia - Alicante)	Liver, Spleen, Skin (5/5) 100.00% (100.00–100.00)	Real-time PCR	100 (100.00–100.00) *N* total detected = 5
	Galán-Puchades et al. (33)	Spain (Barcelona)	Spleen (29/102) 28.43% (19.68–37.19)	qPCR	28.4 (19.65–37.15) *N* total detected = 29
*Rattus rattus*	Psaroulaki et al. (53)	Cyprus	Blood (17/152) 11.18% (6.17–16.19)	IFAT	11.2 (6.19–16.21) *N* total detected = 17
	Zanet et al. (12)	Italy (Montecristo)	Spleen (11/71) 15.49% (7.08–23.91)	PCR	15.5 (7.08–23.92) *N* total detected = 11
	Navea-Pérez et al. (47)	Spain (Granada)	Blood (1/9) 11.11% (0.00–31.64) Bone Marrow (1/9) 11.11% (0.00–31.64) Skin (1/9) 11.11% (0.00–31.64)	PCR-ELISA	33.3 (2.51–64.09) *N* total detected = 3
			Liver (2/9) 22.22% (0.00–49.38)	Microscopy	22.2 (0.00–49.35) *N* total detected = 2)
	Tsakmakidis et al. (59)	Greece (Macedonia)	Liver or Spleen (3/12) 25.00% (0.50–49.50)	Real-time PCR	25 (0.50–49.50) *N* total detected = 3
			Liver or Spleen (0/12) 0.00% (0.00–0.00)	Microscopy	
*Sciurus vulgaris*	Alcover et al. (13)	Spain (Catalonia)	Liver (3/25) 12.00% (0.00–24.74) Spleen (3/25) 12.00% (0.00–24.74) Skin (2/12) 16.67% (0.00–37.75)	qPCR	20 (4.32–35.68) *N* total detected = 5

*Ref., Reference; N, number; CI, Confidence Interval; PCR, Polymerase chain reaction; ELISA, Enzyme-Linked ImmunoSorbent Assay; H.E., hematoxylin-eosin*.

In the studies covered by this review, only some of the criteria outlined by the WHO for defining a species as a reservoir of *Leishmani*a (61) have been met. The reservoir must be sufficiently abundant and long-lived, and there should be continuous contact between the host and vector. Some species of *Phlebotomus* are described as opportunistic, feeding on the most accessible animals (31, 62). Therefore, if the density of rodents is high, they may be expected to have an increased risk of exposure to the bite of the vector. However, given the complexity of the interactions between the different actors of the transmission cycle (protozoan–vector–mammal), the link between the vector and animal host is difficult to prove (63, 64).

In a reservoir population, the prevalence of *Leishmania infantum* should be >20%, which has been found for the following rodent species: *Cricetulus migratorius* (44)*, Mesocricetus auratus* (43, 44), *Mus musculus* (31, 36, 47, 59)*, Mus spretus* (13), *Psammomys obesus* (27), *Psammomys vexillaris* (27), and *Sciurus vulgaris* (13). The house mouse (*M. musculus*), which is native to southwestern Asia (65), is an invasive rodent with a dramatic impact on biodiversity, and human health and activities (66). Asian rodents of the genus *Rattus* have been implicated in the emergence and spread of infectious diseases affecting human health (67, 68). In the Mediterranean region, the global prevalence of *Leishmania* infection in the Norway or brown rat (*Rattus norvegicus)*, and the black or roof rat (*Rattus rattus*) is below 20% but not negligible (9.9 and 16.4%, respectively) (Table 3). Both species merit special attention due to their readiness to colonize urban environments worldwide (69), and their serious impact on global health. Among several publications reporting cases of *Leishmania* infection in rodents, a study using molecular analysis in an urban area of Brazil found that 16.7% of *R. norvegicus*, 10% of *M. musculus* and up to 25% of *Cerradomys subflavus*, a species native to Brazil, tested positive for *L. infantum* (70). These results provide evidence that the control of leishmaniosis in urban areas should take into consideration the potential transmission role of rodents, especially those species that live alongside humans.

Another relevant factor is the course of infection, which must be non-pathogenic and long enough to allow parasites to survive a season without transmission. Our review shows that rodents with clinical manifestations of *Leishmania* infection, such as splenomegaly or hepatomegaly, have not been observed in most studies. Finally, parasites must be accessible in the skin or blood of the host in sufficient quantities to be ingested by a sand fly, which was not demonstrated in the reviewed studies. Table 5 details the rank of the median of infection prevalence in the different tissue samples analyzed, which ranges between 13.75% (spleen) and 33.33% (leg hair). In the case of skin samples, the range of infection prevalence is 8.33–66.67%.

The complexity of the transmission cycle is increased by the diversity of the rodent hosts, as this may give rise to a dilution effect. This hypothesis holds that for vector-borne parasites, the presence of less competent host species may reduce the prevalence of infection in the main host (71) and the relative contribution of each rodent species to the cumulative reservoir can differ (14). Therefore, the equilibrium of each stage of this complex system of ecological and epidemiological interactions between different hosts, pathogens and vectors is affected by the density of rodent populations.

In total, the studies included in this review investigated 23 species of rodents. The number of specimens per species varies considerably, ranging from one of *Funambulus pennanti* to 807 of *Apodemus sylvaticus*. In the latter, 13 specimens (1.6%) were found to be infected (IC_95%_ 0.9–2.8). The species with the highest number of infected specimens was *Rattus norvegicus*: 80 out of 659 animals (9.9%) (IC_95%_ 12.1–14.9). The highest rate of infection was found in *Mesocricetus auratus*, but only four specimens were analyzed, three testing positive (75%) (IC_95%_ of 67.7%). These results indicate that there is still insuficient data available to define which species may have a significant impact on the transmission cycle of the parasite.

## Conclusion

The detection of *Leishmania* infection in rodents, regardless of the species, suggests that these animals may contribute to maintaining the life cycle of *L. infantum*. Further, studies employing xenodiagnosis should shed more light on this role. Additionally, experimental and analytical studies are necessary to evaluate which type of sample and technique are the most suitable to detect the infection. Despite the challenging nature of rodent control, more information about this zoonotic parasite carried by rodent populations in the Mediterranean basin is required to develop suitable surveillance plans and intervention strategies.

## Author Contributions

MA and RF designed the research study and wrote the manuscript. MA, MR, and RF contributed with data analysis and interpretation. All authors read and approved the final version of the manuscript.

## Conflict of Interest

The authors declare that the research was conducted in the absence of any commercial or financial relationships that could be construed as a potential conflict of interest. The handling editor declared a shared affiliation with the authors MA, MR, and RF at time of review.

## Publisher's Note

All claims expressed in this article are solely those of the authors and do not necessarily represent those of their affiliated organizations, or those of the publisher, the editors and the reviewers. Any product that may be evaluated in this article, or claim that may be made by its manufacturer, is not guaranteed or endorsed by the publisher.
