# Supplementary material for: Leishmaniosis in Rodents Caused by Leishmania infantum: A Review of Studies in the Mediterranean Area
Source: Front Vet Sci. 2021 Aug 6;8:702687. doi: 10.3389/fvets.2021.702687 (PMC8377756; doi:10.3389/fvets.2021.702687)
Supplement: Supplementary file 1 [file Data_Sheet_1.DOC]

| S1.- Species and number of specimens studied in each country between 1995 and 2020 | | | | | | | | | | |
| --- | --- | --- | --- | --- | --- | --- | --- | --- | --- | --- |
| **Species** | **Country (number studied)** | | | | | | | | | |
|  | **Algeria** | **Cyprus** | **Spain** | **Greece** | **Iran** | **Morocco** | **Portugal** | **Tunisia** | **Turkey** | **Total** |
| *Apodemus sylvaticus* |  |  | 155 |  | 203 | 17 |  |  | 432 | 807 |
| *Cricetulus migratorius* |  |  |  |  | 36 |  |  |  |  | 36 |
| *Funambulus pennanti* |  |  |  |  | 1 |  |  |  |  | 1 |
| *Gerbilus campestri* |  |  |  |  |  | 4 |  |  |  | 4 |
| *Lemniscomys barbarus* |  |  |  |  |  | 2 |  |  |  | 2 |
| *Mastomys erythroleucus* |  |  |  |  |  | 4 |  |  |  | 4 |
| *Microtus arvalis* |  |  |  |  | 18 |  |  |  |  | 18 |
| *Mesocricetus auratus* |  |  |  |  | 4 |  |  |  |  | 4 |
| *Meriones crassus* |  |  |  |  | 48 |  |  |  |  | 48 |
| *Meriones hurrianae* |  |  |  |  | 28 |  |  |  |  | 28 |
| *Meriones libycus* |  |  |  |  | 461 | 6 |  |  |  | 467 |
| *Mus musculus* |  |  | 23 | 66 | 154 | 50 | 27 |  |  | 320 |
| *Meriones persicus* |  |  |  |  | 107 |  |  |  |  | 107 |
| *Meriones shawi* |  |  |  |  |  | 11 |  |  |  | 11 |
| *Mus spretus* |  |  | 58 |  |  | 14 |  |  |  | 72 |
| *Nesokia indica* |  |  |  |  | 26 |  |  |  |  | 26 |
| *Psammomys obesus* | 3 |  |  |  |  |  |  | 43 |  | 46 |
| *Psammomys vexillaris* |  |  |  |  |  |  |  | 29 |  | 29 |
| *Rattus norvegicus* |  | 342 | 106 | 35 | 164 | 9 | 3 |  |  | 659 |
| *Rhombomys opimus* |  |  |  |  | 591 |  |  |  |  | 591 |
| *Rattus rattus* |  | 152 | 29 | 12 | 30 | 80 |  |  |  | 303 |
| *Sciurus vulgaris* |  |  | 25 |  |  |  |  |  |  | 25 |
| *Tatera indica* |  |  |  |  | 35 |  |  |  |  | 35 |
| **Total** | **3** | **494** | **396** | **113** | **1906** | **197** | **30** | **72** | **432** | **3643** |
