# Supplementary material for: Leishmaniosis in Rodents Caused by Leishmania infantum: A Review of Studies in the Mediterranean Area
Source: Front Vet Sci. 2021 Aug 6;8:702687. doi: 10.3389/fvets.2021.702687 (PMC8377756; doi:10.3389/fvets.2021.702687)
Supplement: Supplementary file 2 [file Data_Sheet_2.DOC]

| S2.- Species and number of infected specimens in each country between 1995 and 2020 | | | | | | | | | | |
| --- | --- | --- | --- | --- | --- | --- | --- | --- | --- | --- |
| **Species** | **País (n infectado)** | | | | | | | | | |
|  | **Algeria** | **Cyprus** | **Spain** | **Greece** | **Iran** | **Morocco** | **Portugal** | **Tunisia** | **Turkey** | **Total** |
| *Apodemus sylvaticus* |  |  | 8 |  | 0 | 0 |  |  | 5 | 13 |
| *Cricetulus migratorius* |  |  |  |  | 3 |  |  |  |  | 3 |
| *Funambulus pennanti* |  |  |  |  | 0 |  |  |  |  | 0 |
| *Gerbilus campestri* |  |  |  |  |  | 0 |  |  |  | 0 |
| *Lemniscomys barbarus* |  |  |  |  |  | 0 |  |  |  | 0 |
| *Mastomys erythroleucus* |  |  |  |  |  | 0 |  |  |  | 0 |
| *Microtus arvalis* |  |  |  |  | 0 |  |  |  |  | 0 |
| *Mesocricetus auratus* |  |  |  |  | 3 |  |  |  |  | 3 |
| *Meriones crassus* |  |  |  |  | 0 |  |  |  |  | 0 |
| *Meriones hurrianae* |  |  |  |  | 0 |  |  |  |  | 0 |
| *Meriones libycus* |  |  |  |  | 5 | 0 |  |  |  | 5 |
| *Mus musculus* |  |  | 18 | 16 | 1 | 9 | 9 |  |  | 53 |
| *Meriones persicus* |  |  |  |  | 7 |  |  |  |  | 7 |
| *Meriones shawi* |  |  |  |  |  | 0 |  |  |  | 0 |
| *Mus spretus* |  |  | 14 |  |  | 0 |  |  |  | 14 |
| *Nesokia indica* |  |  |  |  | 0 |  |  |  |  | 0 |
| *Psammomys obesus* | 0 |  |  |  |  |  |  | 18 |  | 18 |
| *Psammomys vexillaris* |  |  |  |  |  |  |  | 8 |  | 8 |
| *Rattus norvegicus* |  | 19 | 34 | 8 | 18 | 1 | 0 |  |  | 80 |
| *Rhombomys opimus* |  |  |  |  | 61 |  |  |  |  | 61 |
| *Rattus rattus* |  | 17 | 3 | 3 | 1 | 6 |  |  |  | 30 |
| *Sciurus vulgaris* |  |  | 5 |  |  |  |  |  |  | 5 |
| *Tatera indica* |  |  |  |  | 2 |  |  |  |  | 2 |
| **Total** | **0** | **36** | **82** | **27** | **101** | **16** | **9** | **26** | **5** | **302** |
